# COVID19 antibody detection using lateral flow assay tests in a cohort of convalescent plasma donors

**DOI:** 10.1186/s13104-020-05212-0

**Published:** 2020-08-06

**Authors:** Brett Ragnesola, Daniel Jin, Christopher C. Lamb, Beth H. Shaz, Christopher D. Hillyer, Larry L. Luchsinger

**Affiliations:** 1 New York Blood Center Lindsley F. Kimball Research Institute, 310 E 67th Street, New York, NY 10065 USA; 2 BioSolutions Services, 92 Irving Avenue, Englewood Cliffs, NJ 07632 USA; 3 Department of Management and Entrepreneurship, Silberman College of Business, Fairleigh Dickinson University, Teaneck, NJ USA; 4 Weatherhead School of Management, Case Western Reserve University, Cleveland, OH USA

**Keywords:** Covid-19, Antibody testing, Convalescent plasma

## Abstract

**Objective:**

COVID19 has caused a global and ongoing pandemic. The need for population seroconversion data is apparent to monitor and respond to the pandemic. Using a lateral flow assay (LFA) testing platform, the seropositivity in 63 New York Blood Center (NYBC) Convelescent Plasma (CP) donor samples were evaluated for the presence of COVID19 specific IgG and IgM.

**Results:**

CP donors showed diverse antibody result. Convalescent donor plasma contains SARS-CoV-2 specific antibodies. Weak antibody bands may identify low titer CP donors. LFA tests can identify antibody positive individuals that have recovered from COVID19. Confirming suspected cases using antibody detection could help inform the patient and the community as to the relative risk to future exposure and a better understanding of disease exposure.

## Introduction

Severe acute respiratory syndrome coronavirus 2 (SARS-CoV-2) has caused over 4012,000 infections and  > 32,000 deaths in New York State alone [1]. Due to delay in testing and asymptomatic infections the true number of cases are unknown. Few reports have characterized the prevalence of seroconversion in community populations [2, 3]. Seroconversion, the process in which a patient accumulates antigen-specific antibodies against an epitope, is the first step towards the development of adaptive immunity against pathogens. Although it is not an assurance of protection against future infections, positive seroconversion is an informative measure of previous viral infectivity within the population. To assess the seroconversion of a community, antibody testing with high sensitivity and specificity that is also easily available is necessary.

However, a crucial step in understanding the test characteristics is to ensure the assay detects antibodies in individuals with a previous documented disease. One study suggests that 75% of patients with a confirmed PCR test had a positive antibody IgG and 20% were weakly positive [4]. Another study showed 100% seroconversion in COVID19 patients and three patterns of IgM and IgG responses: synchronous seroconversion of IgG and IgM, IgM seroconversion earlier than that of IgG, and IgM seroconversion later than that of IgG [3]. In addition, assay characteristics such as antigen target (nucleocapsid and/or spike glycoprotein), total (IgG and IgM) versus IgG only, and their sensitivity and specificity are important in defining seroconversion rates [5]. Thus, more studies with various antibody tests are needed to understand seroconversion of an infected population.

In response to this need for antibody testing, a lateral flow assay (LFA) was developed to provide rapid point of care diagnostic testing of COVID19 antibodies. The LFA test is able to detect specific SARS-CoV-2 antibodies and differentiate between IgG and IgM immunoglobin classes in a rapid, point of care test using either whole blood, plasma or serum [6]. The test principle is based on the receptor-binding domain (RBD) of the spike and nucleocapsid proteins. The cassette has both a dye pad which contains colloidal gold coupled with Recombinant 2019-novel coronavirus nucleocapsid protein and a dye pad which contains colloidal gold coupled with Recombinant 2019-novel coronavirus Spike Protein (Si Subunit). Thus, LFAs are potentially useful assays that require low sample input and minimum processivity. In this study, we report the sensitivity and specificity of Clungene^®^ SARS-CoV-2 IgG/IgM Rapid Test Cassettes in determining the presence of binding antibodies in convalescent plasma (CP) donor samples with previously documented COVID19.

## Main text

### Methods

Convalescent donor plasma was collected by the New York Blood Center (NYBC) with written consent from patients in accordance with NYBC Institutional Review Board protocols. All donors had self-reported documented COVID19 disease by positive SARS-CoV-2 RT-PCR test (manufacturer and documentation not provided from referring institution of CP donors), had complete resolution of symptoms at least 14 days prior to donation, and otherwise met all criteria for donating blood consistent with FDA’s policy on the Collection of COVID-19 Convalescent Plasma [1]. As a negative control, fresh frozen plasma was used that was collected prior to the beginning of the epidemic. Clungene^®^ SARS-CoV-2 (COVID-19) IgG/IgM Rapid Test Cassettes were used to determine the presence of SARS-CoV-2-specific IgG and IgM. The manufacturer of the Cassette (Hangzhou Clongene Biotech Co., Ltd., Hangzhou, China) validated this immunoassay for the qualitative detection of IgG and IgM antibodies to SARS-CoV-2 and these data were submitted to FDA as part of their Emergency Use Authorization [7].

**Fig. 1:**
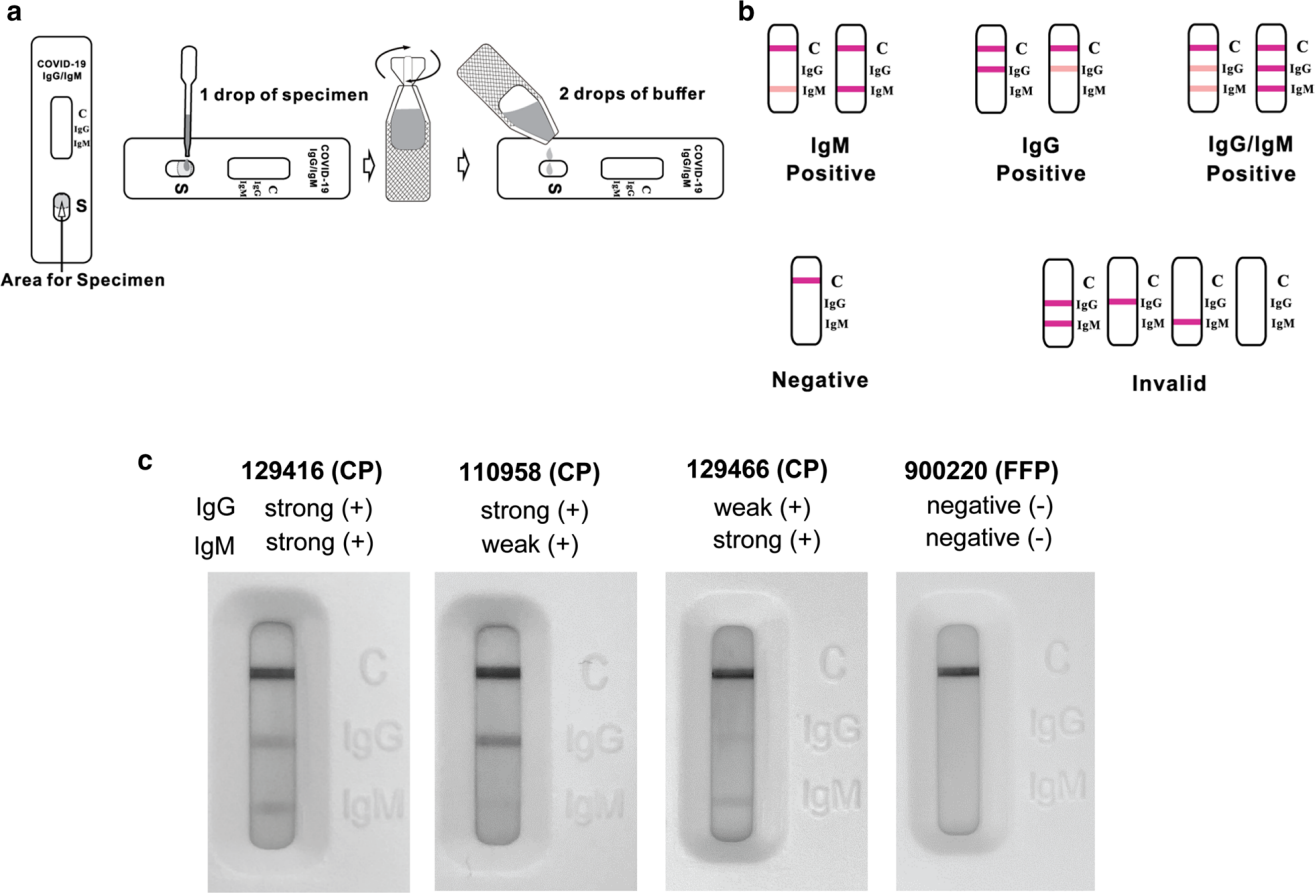
**a** Procedural schematic for CLUNGENE^®^ Immunoassay. One drop is equal to ~ 20 uL. **b** Visual interpretation guide for assays. **c** Representative convalescent donor plasma (CP) or frozen fresh plasma (FFP) assay result images

To perform assays, 20 mL of human plasma was applied to the sample pad followed by two drops of proprietary running buffer. Tests were analyzed after 15 min. Following incubation, high resolution images were taken of detection zone and saved as JPEG for reference and analysis. Positive and negative IgG/IgM band determinations were made by visual inspection with accordance to manufacturer instructions (Fig. 1a, b). All tests were performed under a NYBC IRB approved protocol using four independently trained operators.

### Results

**Fig. 2:**
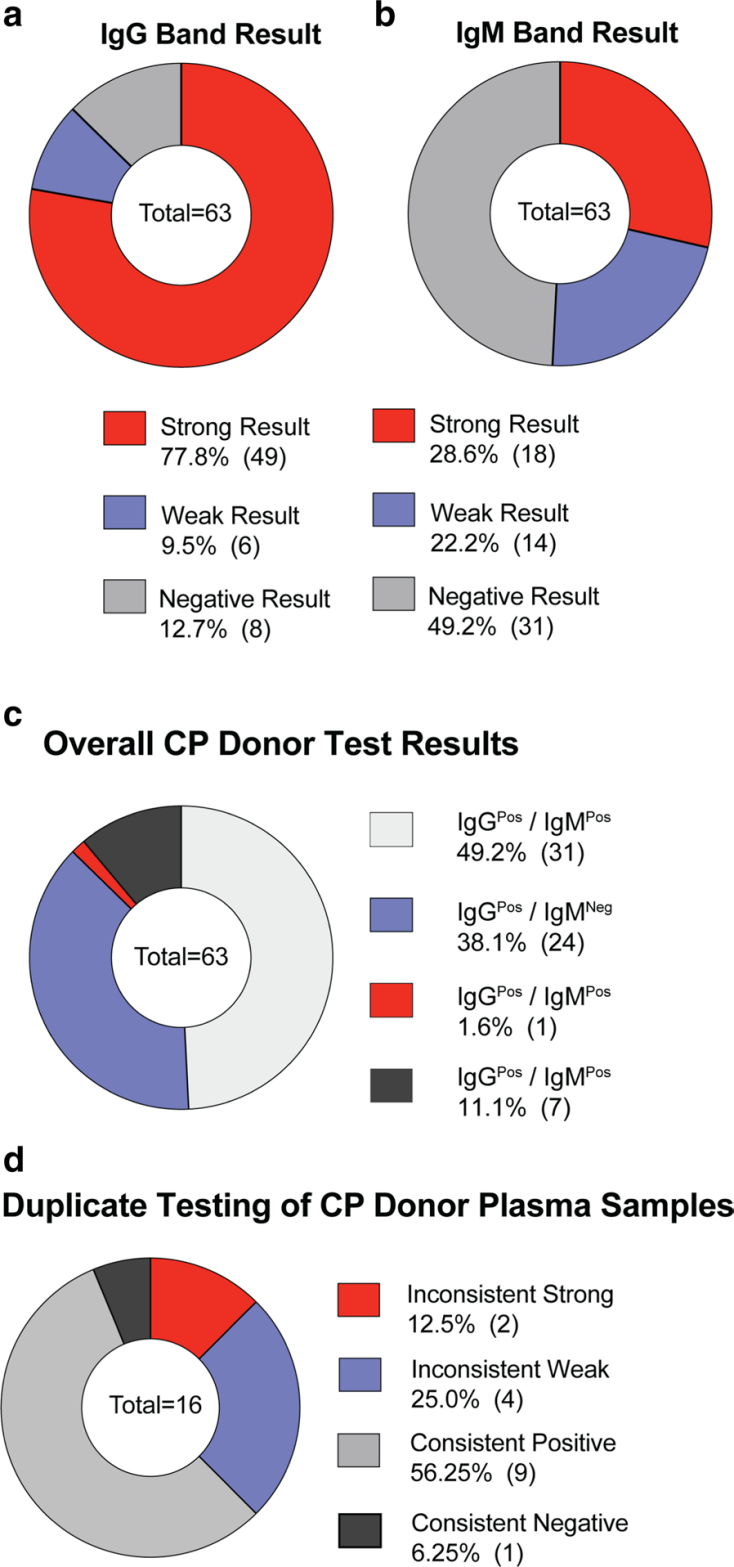
**a** Frequency of IgG assay results from CP donor samples. **b** Frequency of IgM assay results from CP donor samples. **c** Overall CP donor test result. **d** Frequency of assay result duplication using identical CP donor samples

Convalescent donor plasma contains SARS-CoV-2 specific antibodies. Using CP donors as a prospectively positive population, we tested 63 NYBC CP donor samples for the presence of SARS-CoV-2 specific IgG and IgM. CP donors showed diverse antibody result profiles with the LFA test, including strong and weak bands as compared to FFP negative control (Fig. 1c, Table 1). All samples yielded an interpretable result with no invalid results. Overall, 88.9% (56/63) of CP donors were considered positive. 87.3% (55/63) of CP donors were positive for IgG and 50.8% (32/63) of CP donors were positive for IgM (Fig. 2a, b).

**Table 1:** Compilation of LFA testing results

Sample#	Original/duplicated	Experimenter	Sample ID	IgG result	IgM result
1	Original	A	73,573	Weak+	Negative
2	Original	B	96,138	Negative	Negative
3	Original	C	96,245	Strong+	Negative
4	Original	B	110,766	Strong+	Strong+
5	Original	A	110,773	Strong+	Negative
6	Original	B	110,781	Strong+	Strong+
7	Original	B	110,782	Strong+	Negative
8	Original	A	110,788	Strong+	Negative
9	Original	A	110,790	Strong+	Weak+
10	Original	A	110,802	Strong+	Weak+
11	Original	A	110,810	Strong+	Weak+
12	Original	A	110,811	Weak+	Weak+
13	Original	C	110,958	Strong+	Weak+
14	Original	C	110,973	Strong+	Strong+
15	Original	C	110,984	Strong+	Negative
16	Original	B	110,988	Strong+	Negative
17	Original	C	111,846	Strong+	Strong+
18	Original	B	111,847	Strong+	Weak+
19	Original	C	111,848	Strong+	Strong+
20	Original	C	111,857	Strong+	Negative
21	Original	C	116,229	Strong+	Strong+
22	Original	B	117,031	Strong+	Weak+
23	Original	B	117,032	Strong+	Strong+
24	Original	B	117,055	Negative	Negative
25	Original	B	117,072	Weak+	Negative
26	Original	A	117,102	Strong+	Weak+
27	Original	B	117,131	Negative	Negative
28	Original	C	117,707	Strong+	Negative
29	Original	C	127,010	Strong+	Negative
30	Original	C	127,161	Negative	Negative
31	Original	C	127,168	Negative	Negative
32	Original	C	127,171	Strong+	Negative
33	Original	C	127,179	Strong+	Negative
34	Original	D	129,402	Strong+	Strong+
35	Original	A	129,404	Strong+	Strong+
36	Original	D	129,405	Strong+	Negative
37	Original	A	129,408	Negative	Negative
38	Original	B	129,412	Strong+	Weak+
39	Original	B	129,414	Strong+	Weak+
40	Original	B	129,416	Strong+	Strong+
41	Original	A	129,420	Strong+	Strong+
42	Original	D	129,427	Strong+	Negative
43	Original	A	129,437	Weak+	Strong+
44	Original	A	129,455	Strong+	Strong+
45	Original	A	129,466	Weak+	Strong+
46	Original	A	129,471	Strong+	Strong+
47	Original	A	129,483	Strong+	Weak+
48	Original	B	129,491	Strong+	Strong+
49	Original	B	129,790	Strong+	Negative
50	Original	A	129,845	Weak+	Negative
51	Original	B	129,857	Strong+	Strong+
52	Original	C	129,884	Strong+	Negative
53	Original	C	129,900	Strong+	Negative
54	Original	C	97,591	Strong+	Negative
55	Original	B	97,594	Strong+	Weak+
56	Original	C	97,595	Strong+	Strong+
57	Original	C	97,643	Strong+	Negative
58	Original	B	97,723	Strong+	Weak+
59	Original	B	111,538	Strong+	Negative
60	Original	B	111,584	Negative	Negative
61	Original	C	117,001	Strong+	Negative
62	Original	C	129,298	Strong+	Negative
63	Original	B	129,349	Negative	Weak+
FFP 1	Original	C	FFP-181,484	Negative	Strong+
FFP 2	Original	A	FFP-203,529	Negative	Weak+
FFP 3	Original	A	FFP-222,235	Negative	Negative
FFP 4	Original	A	FFP-222,252	Negative	Negative
FFP 5	Original	A	FFP-222,353	Negative	Negative
FFP 6	Original	A	FFP-222,427	Negative	Negative
FFP 7	Original	A	FFP-222,604	Negative	Negative
FFP 8	Original	A	FFP-222,633	Negative	Negative
FFP 9	Original	A	FFP-900,220	Negative	Negative
FFP 10	Original	A	FFP-906,227	Negative	Negative
4	Duplicated	D	110,766	Strong+	Strong+
4	Duplicated	B	110,766	Strong+	Strong+
6	Duplicated	D	110,781	Strong+	Weak+
6	Duplicated	B	110,781	Strong+	Strong+
7	Duplicated	D	110,782	Strong+	Negative
7	Duplicated	B	110,782	Strong+	Negative
22	Duplicated	D	117,031	Negative	Negative
22	Duplicated	B	117,031	Strong+	Weak+
23	Duplicated	D	117,032	Strong+	Negative
23	Duplicated	B	117,032	Strong+	Strong+
24	Duplicated	D	117,055	Negative	Negative
24	Duplicated	B	117,055	Negative	Negative
25	Duplicated	D	117,072	Negative	Negative
25	Duplicated	B	117,072	Weak+	Negative
34	Duplicated	D	129,402	Strong+	Strong+
34	Duplicated	B	129,402	Strong+	Weak+
36	Duplicated	D	129,405	Strong+	Negative
36	Duplicated	B	129,405	Strong+	Negative
38	Duplicated	D	129,412	Strong+	Negative
38	Duplicated	B	129,412	Strong+	Weak+
39	Duplicated	D	129,414	Strong+	Negative
39	Duplicated	B	129,414	Strong+	Weak+
40	Duplicated	D	129,416	Strong+	Strong+
40	Duplicated	B	129,416	Strong+	Strong+
42	Duplicated	D	129,427	Strong+	Negative
42	Duplicated	B	129,427	Negative	Negative
48	Duplicated	D	129,491	Strong+	Weak+
48	Duplicated	B	129,491	Strong+	Strong+
49	Duplicated	D	129,790	Strong+	Negative
49	Duplicated	B	129,790	Strong+	Negative
51	Duplicated	D	129,857	Strong+	Strong+
51	Duplicated	B	129,857	Strong+	Strong+

With regard to negative samples, 11.1% (7/63) of CP donors were IgG^Neg^ and IgM^Neg^, 1.6% (1/63) were IgG^Neg^ and IgM^Pos^ and 38.1% (24/63) were IgG^Pos^ and IgM^Neg^ (Fig. 2c). In contrast, all FFP samples were IgG^Neg^and 80% (8/10) were IgM^Neg^. These data suggest that LFA tests possess a high degree of sensitivity (87.3% IgG, 50.8% IgM) and specificity (100.0% IgG, 80.0% IgM) for detecting SARS-CoV-2 specific antibodies. Given all CP donors were collected more than 14 days since date of last symptom, when the IgM tests would have performed, it is not surprising that the IgM results were low since IgM immunoglobins likely develop early in response to infection [8].

Weak antibody bands may identify low titer CP donors. Recent studies suggest that a significant percentage of convalescent individuals may have low SARS-CoV-2 IgG or IgM titers [4, 9]. We also inferred from conducting LFA assays that potential differences in antibody levels may occur in the CP donor population. However, LFA tests are designed to perform qualitative, and not quantitative, analysis as stated in the manufacturer’s instructions. Nevertheless, to document this phenomenon, trained experimenters subjectively delineated positive results as ‘strong’ or ‘weak’ relative to the band intensity produced by each CP donor sample (Fig. 1c).

To confirm reproducibility, we re-tested random samples (n = 16) to explore whether CP donor samples could provide reproducible results (Fig. 2d, Table 1). Between replicates of paired results, 56.25% (9/16) of samples were consistently positive, 6.25% (1/16) was consistently negative, and 37.5% (6/16) were inconsistent. With regard to inconsistency, these bands were almost always visually weak (4/6). These data suggest that certain CP donors may have low levels of SARS-CoV-2 antibodies and may account for inconsistency between results, while data available from the manufacturer did report any difference related to batches (n = 3), operators (n = 2), runs (n = 2) or time (15 days).

### Discussion

Our study analyzed blood samples from COVID19 convalescent plasma donors to determine whether antibodies are detected using a LFA in this population. We found that the CLUNGENE^®^ SARS-COV-2 VIRUS (COVID-19) IgG/IgM LFA test possesses high sensitivity and specificity for COVID19 antibodies. The LFA test was easy to use with properly trained staff. Results were interpretable within 15 min and the internal procedural control confirmed that sufficient specimen volume, adequate membrane wicking and correct procedural technique were used. Since documented positive PCR tests or comparison to other antibody testing platforms were unavailable, we cannot state that the 7 negative donors in fact were infected or if they have antibody. Even if CP donor infection data were available, it may also be possible, and is probable, that some CP donors produced low amounts of antibodies that is specific to the immunological response unique to each individual, thus, below the detection limit of the LFA. The IgG results are consistent with the manufacturer’s 97.4% clinical performance data which showed positive IgG agreement with known positive RT-PCR test. The IgM results are consistent with recently published data which shows that IgM can persist more than 23 days after symptom onset and can be earlier, synchronous or later than IgG.

### Conclusions

Most (90%) COVID19 convalescent donors seroconverted, demonstrating the potential of LFA tests to identify antibody positive individuals that have recovered from COVID19. Confirming suspected SARS-CoV-2 cases using antibody detection at the point of care could help inform the patient and the community as to the relative risk to future SARS-CoV2- exposure and a better understanding of disease exposure. However, a coherent description of the immunological response and antiviral antibody activity (i.e. neutralizing activity) is warranted to definitively use antibody presence to prognose future disease potential [10]. This study highlights the relevance of serological testing to support accurate estimates of the extent of the COVID-19 pandemic and the potential to assess a patient response to SARS-CoV-2 infection using antibody detection.

## Limitations

Our study has several limitations, including but not limited to:

- Samples were not tested for virus neutralization; therefore neutralizing activities of the detected IgG antibodies are not known.
- The small sample size of patients and the absence of documented PCR test results makes it difficult to determine the relationship between antibody response and clinical course.
- More detailed investigation of the reproducibility of the test is warrented.

## Data Availability

The datasets used and/or analysed during the current study are available from the corresponding authors on reasonable request.

## Abbreviations

LFA: Lateral flow assay
SARS-CoV-2 aka COVID19: Severe acute respiratory syndrome coronavirus 2
CP: Convalescent plasma
IgG: Immunoglobulin G
IgM: Immunoglobulin M
NYBC: New York Blood Center
PCR: Polymerase chain reaction
